# Assessment of bilirubin levels in patients with cirrhosis via forehead, sclera and lower eyelid smartphone images

**DOI:** 10.1371/journal.pdig.0000357

**Published:** 2023-10-06

**Authors:** Miranda Nixon-Hill, Rajeshwar P. Mookerjee, Terence S. Leung

**Affiliations:** 1 Department of Medical Physics and Biomedical Engineering, University College London, United Kingdom; 2 Institute for Liver and Digestive Health, University College London, United Kingdom; National Tsing-Hua University: National Tsing Hua University, TAIWAN

## Abstract

One of the key biomarkers evaluating liver disease progression is an elevated bilirubin level. Here we apply smartphone imaging to non-invasive assessment of bilirubin in patients with cirrhosis. Image data was processed using two different approaches to remove variation introduced by ambient conditions and different imaging devices—a per-image calibration using a color chart in each image, and a two-step process using pairs of flash/ no-flash images to account for ambient light in combination with a one-time calibration. For the first time, results from the forehead, sclera (white of the eye) and lower eyelid were compared. The correlation coefficients between the total serum bilirubin and the predicted bilirubin via the forehead, sclera and lower eyelid were 0.79, 0.89 and 0.86 (all with p<0.001, n = 66), respectively. Given the simpler image capture for the sclera, the recommended imaging site for this patient cohort is the sclera.

## Author summary

Cirrhosis patients have decreased liver function, which affects its ability to remove bilirubin in the blood leading to jaundice. To monitor cirrhosis progression, patients’ bilirubin levels are regularly measured by blood tests, which require clinical expertise and specialist equipment. In this work, we have investigated the feasibility of adopting an alternative approach based on a smartphone and its camera. The yellow-colored bilirubin causes discoloration in the patient’s skin and sclera, making them appear yellow. It is generally perceived that the degree of yellowness is indicative of the bilirubin level. We have developed smartphone imaging techniques to minimise the effects of ambient lighting, calibrate for individual camera’s characteristics, and predict the bilirubin level based on photos of the forehead, sclera and lower eyelid. Our results show that there are strong correlations between the total serum bilirubin measure by blood tests and the predicted bilirubin estimated from photos of the forehead, sclera and lower eyelid, showing the potential of further developing this technique into a non-invasive diagnostic technique for monitoring cirrhosis patients.

## Introduction

Liver disease is the third most common cause of working-age premature death in the UK [1]. Whilst mortality rates have greatly improved for many chronic diseases, the mortality rate for liver disease in the UK increased by 400% between 1970 and 2010 [1]. There is an increasing need to obtain cost-effective and non-invasive measures to monitor cirrhosis progression. Here, we focus on assessing the bilirubin level, which rises when the diseased liver becomes less able to process this normal red blood cell breakdown product. Rising bilirubin leads to visible yellowing of the skin and whites of the eyes (sclera), termed jaundice.

Unlike the standard invasive blood test, this visible yellowing can be used to assess the bilirubin level in a non-invasive manner. The skin, specifically the forehead, is a common choice, as it is flat, readily available and is relatively matte so does not suffer from issues of reflection [2]. However, the presence of melanin in the skin means that every person will have a different baseline skin color, depending on their ethnicity. The sclera is another typical choice as it is free from melanin—the human sclera is always white in healthy individuals [3, 4]. There are drawbacks of using the sclera which include a small available area and the high occurrence of reflections owing to the layer of tear covering the eye. Another option is to use the inside of the lower eyelid (palpebral conjunctiva), which is far less affected by melanin than the skin. Unlike the skin and sclera, viewing the inside of the lower eyelid requires a slightly uncomfortable pulling down of the eyelid. The lower eyelid is also prone to reflection, for the same reason as the sclera.

Rather than using visual inspection, as is standard clinical practice, imaging is used to assess the discoloration. This allows a shift from qualitative to quantitative assessment. Smartphone imaging lends itself as an excellent tool for clinical adoption, since smartphones are becoming cheaper, more powerful and ubiquitous with over 8 billion subscriptions by 2019 [5]. The image quality continues to improve and via apps, smartphones have the ability to carry out on-board processing, thereby providing real-time results.

In order to obtain quantitative results, it is necessary to handle image data carefully. Without processing, results will depend on both the lighting conditions and the device used [6]. Due to varying camera spectral sensitivity, different devices will record different colors for identical scenes. Since gathering the patient image data required to produce models is extremely time consuming, repeating this process with different devices is not desirable. The image data should instead be carefully processed to produce device and capture condition independent results.

Here, we investigate different imaging sites to assess bilirubin in cirrhosis patients. We first demonstrate the compatibility of bilirubin predictions from two different devices, using a previously published color correction method [6]. We then compare results based on the skin, sclera and lower eyelid to choose the optimal site for bilirubin assessment in cirrhosis patients, covering the wide range of bilirubin values encountered in this patient cohort.

## Methods

### Image capture and processing—forehead

**Fig 1:**
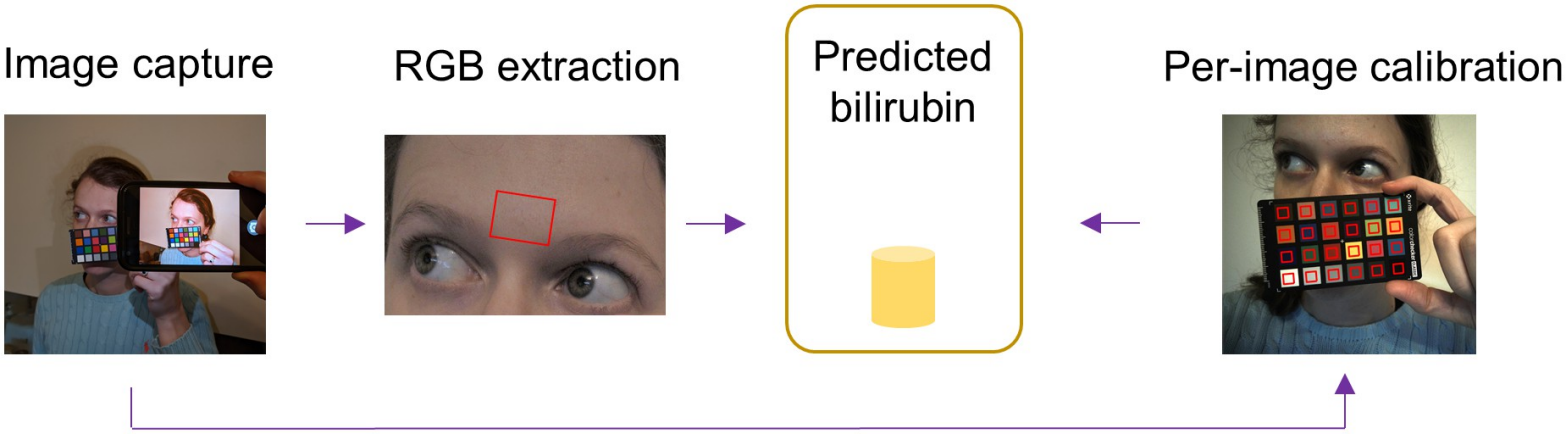
Per-image processing method. Images of the face are collected under ambient conditions, including a color chart in each shot. A per-image color mapping is developed from RGB to XYZ using the color chart and applied to the average RGB value for the forehead region of interest. The predicted bilirubin is then calculated based on the xy chromaticities.

Images of the face were collected, and the images were corrected for ambient lighting changes and device dependence using a per-image calibration method. A standard way to do this is to include a color chart in each image [7, 8], for example the X-Rite ColorChecker Classic. A schematic of the method is shown in Fig 1. A 3 × 3 color mapping from device RGB to the standard device-independent XYZ space can then be developed using the chart, and applied to data from that image alone. Even when under relatively spatially homogeneous ambient lighting, there is likely to be a variation in the illumination intensity across the chart. Finlayson et al proposed using an iterative alternating least squares (ALS) method—the shading across the chart is estimated keeping the color mapping fixed, then the color mapping is estimated using the shading estimate, and the process repeated until convergence on ***M*** [9]. The median RGB value for the forehead skin ROI (carefully chosen to avoid specular reflection) from single ambient images were extracted and the per-image color mapping applied to move to XYZ space,

$$\begin{array}{c} {}[\text{XYZ}]=[\text{RGB}]M \end{array}$$ (1)

The resulting XYZ values are independent of the device and ambient conditions. The final stage is to link these color values to bilirubin levels. Chromaticity values are used to avoid issues caused by shading, different exposure times or phone-eye distances across images. Chromaticity is defined as a color channel divided by the sum of the other channels—for example x chromaticity is given by *x* = *X*/(*X* + *Y* + *Z*). The prediction of bilirubin is based on a regression model established between TSB and x and y chromaticities of all patients [10]—i.e. predicted bilirubin = *p* × *x* + *q* × *y* + *r*, where *x* and *y* were chromaticities calculated from the RGB pixel values of the region of interest, and *p*, *q* and *r* were parameters estimated from a linear regression model with *x* and *y* being the independent variables and the measured bilirubin (TSB) being the dependent variable. All samples in the dataset were used to optimise the estimation model, which was then applied to predict bilirubin in all patients. To assess the performance of the estimation model, the correlation between TSB and the predicted bilirubin was calculated and used as the main result.

### Image capture and processing—Sclera and lower eyelid

**Fig 2:**
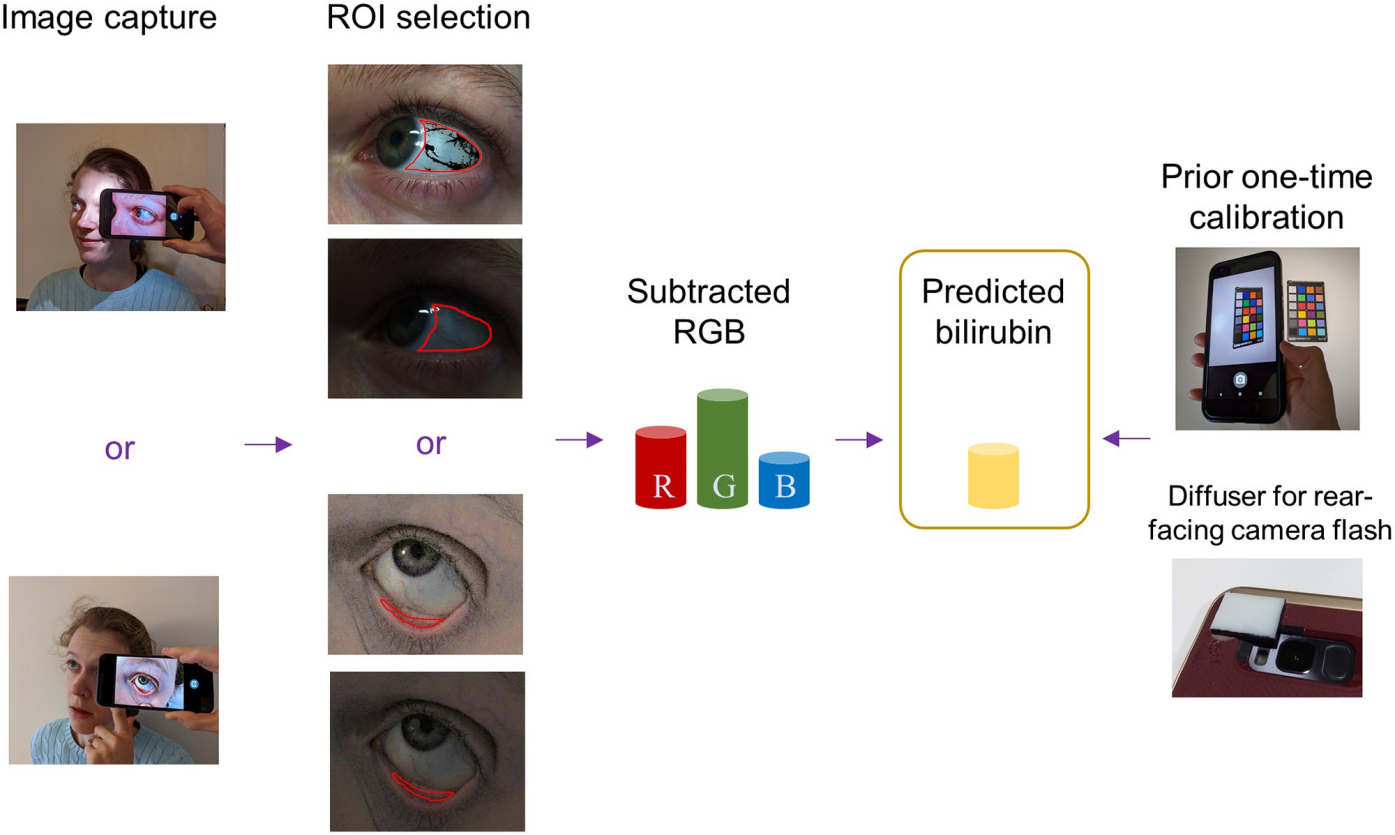
Subtraction and one-time calibration processing method. Flash/ no-flash image pairs are captured of the sclera and lower eyelid, the region of interest is selected and pixels containing blood vessels and specular reflection are discounted from scleral analysis. The average RGB values for the no-flash image are subtracted from the flash RGB values. As a one-time step, a device-specific color mapping from RGB to XYZ is developed using a color chart imaged under the flash illumination of the phone. This color mapping is applied to the subtracted RGB value and the bilirubin prediction then calculated from the xy chromaticites.

An alternative processing method was used for images captured of the sclera and lower eyelid, as described in [6]. This approach avoids the need to include a color chart in each image. The most relevant aspects and improvements for this application are summarised here. An overview of the process is shown in Fig 2. Flash/ no-flash image pairs are captured, and data from the no-flash image subtracted from the flash image. For a given device, the effective illumination is thereby standardised to the flash of the phone alone [6, 10]. The subtracted signal to noise ratio (SSNR) can be used to ensure that there is sufficient difference between flash and no-flash images in the region of interest [6].

When approaching scleral imaging in adults, there are typically many blood vessels and regions of specular reflection which can distort results. We therefore use a simple method to remove the majority of these pixels from the selected region of interest. A test for specular pixels is first carried out based on the presence of very high channel values within the region of interest. If specular pixels are deemed present, a modified and simplified version of [11] is used to remove these: the image is converted to the Hue Saturation Value (HSV) color space, a power law enhancement applied to the V channel and Otsu thresholding carried out on the result [12]. Vessel filtering is then carried out on the resulting pixels in the region of interest by calculating the red chromaticity of the image and using Otsu thresholding. The median can then be calculated for the remaining pixels in the flash and no-flash images, to remove any remaining outliers and minimize the effects of movement artefacts, before finally applying subtraction.

Once the results are standardised for a given phone, the second step is to remove dependence on the device—even variations between handsets of the same model will impact accuracy [13]. Images of a color chart with patches of known colors, such as the X-Rite ColorChecker Classic, are captured under the phone’s flash illumination with no ambient light. A color mapping ***M*** can be developed once and applied to all subtracted data for a given phone, since the effective lighting is standardised to the flash of the phone [6], moving to XYZ space as in (1). It is very important to capture and account for the high level of intensity non-uniformity of the flash across the color chart in order to maintain a high color mapping accuracy [6]. For this reason, the ALS approach described above was used to develop the per-device ***M***s [9]. Using this approach, it is possible to avoid the need for a phone tripod and grey card, typically used to measure the shading field. Instead, the one-time calibration image capture process is simplified through the use of just handheld images of the color chart. As described in the previous section, predicted bilirubin levels can then be generated based on the xy chromaticities [10].

### Clinical data collection

One hundred and three sets of images of patients with cirrhosis were collected at the Royal Free Hospital in London. Ethics for this study was granted as part of an on-going biomarker study approved by the institutional review board (London—Harrow; REC Ref: 08/H0714/8) and all patients provided informed consent. Three types of image pairs were collected. The first focussed on the sclera alone, the second focussed on the lower eyelid, and the third focussed on the forehead alongside an X-Rite ColorChecker Classic chart, which (the same one) was used consistently in all patients.

Images were collected in parallel using two phones—an LG Nexus 5X (Nexus) and a Samsung S8 (S8). The Nexus used the screen turned on white as the “flash” with the front-facing camera. This photo capture mode would be useful for patients conducting self-monitoring. The S8 used the rear-facing camera with the camera flash, where a custom diffuser was used to spread and dim the light (depicted in Fig 2). A custom-developed app was used for image capture on both phones; when opened, the flash turned on to allow acclimatisation, and when capture was requested, a raw flash and no-flash image were saved in quick succession. The total serum bilirubin (TSB) level was also collected via a blood test as part of standard care. Ideally all three image types would have been collected using both phones for each patient image set but this was not always possible in practice. The different numbers of available images for each category of images are reflected in the presented results.

## Results

### Demonstration of processing

To avoid repeating the time-consuming process of capturing clinical data, it is vital that image data is collected in such a way that it can be processed to remove the dependence on both the ambient conditions and the capture device. Here, we demonstrate the importance of fully applying processing for the two methods used in this paper before focussing on comparing the results from different imaging sites for a single phone.

**Fig 3:**
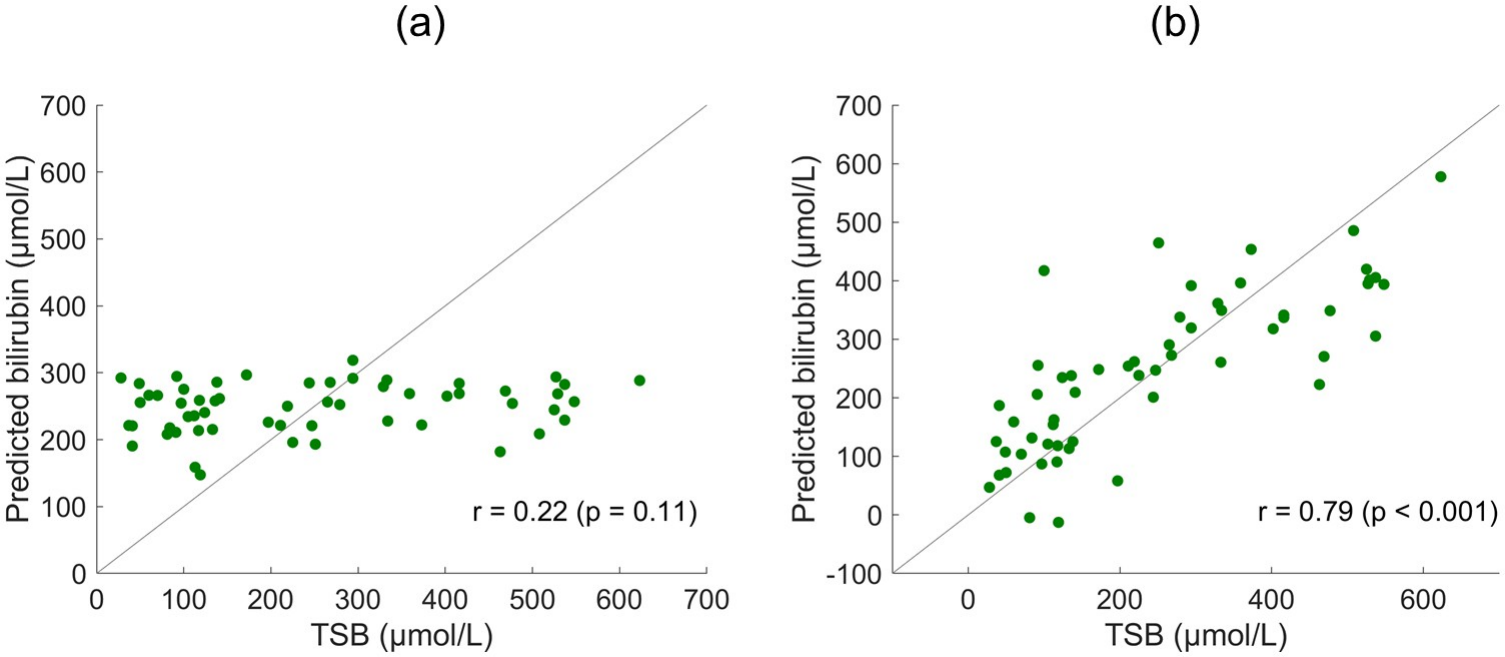
Importance of color correction. Predicted bilirubin levels are shown against the measured total serum bilirubin level (TSB) for the forehead site using S8 phone images without using and with the per-image mappings in (a) and (b) respectively. Pearson linear correlation coefficients are displayed for each plot (n = 57).

Fig 3 presents forehead data for the S8 phone before and after applying the per-image mappings. Without attempting to correct for ambient lighting (or device dependence), no trend is seen. In this case, it is very clear that the full processing is necessary—when using the per-image mappings there is a strong significant correlation observed. Further detail and discussion on the data itself is presented in the next section.

**Fig 4:**
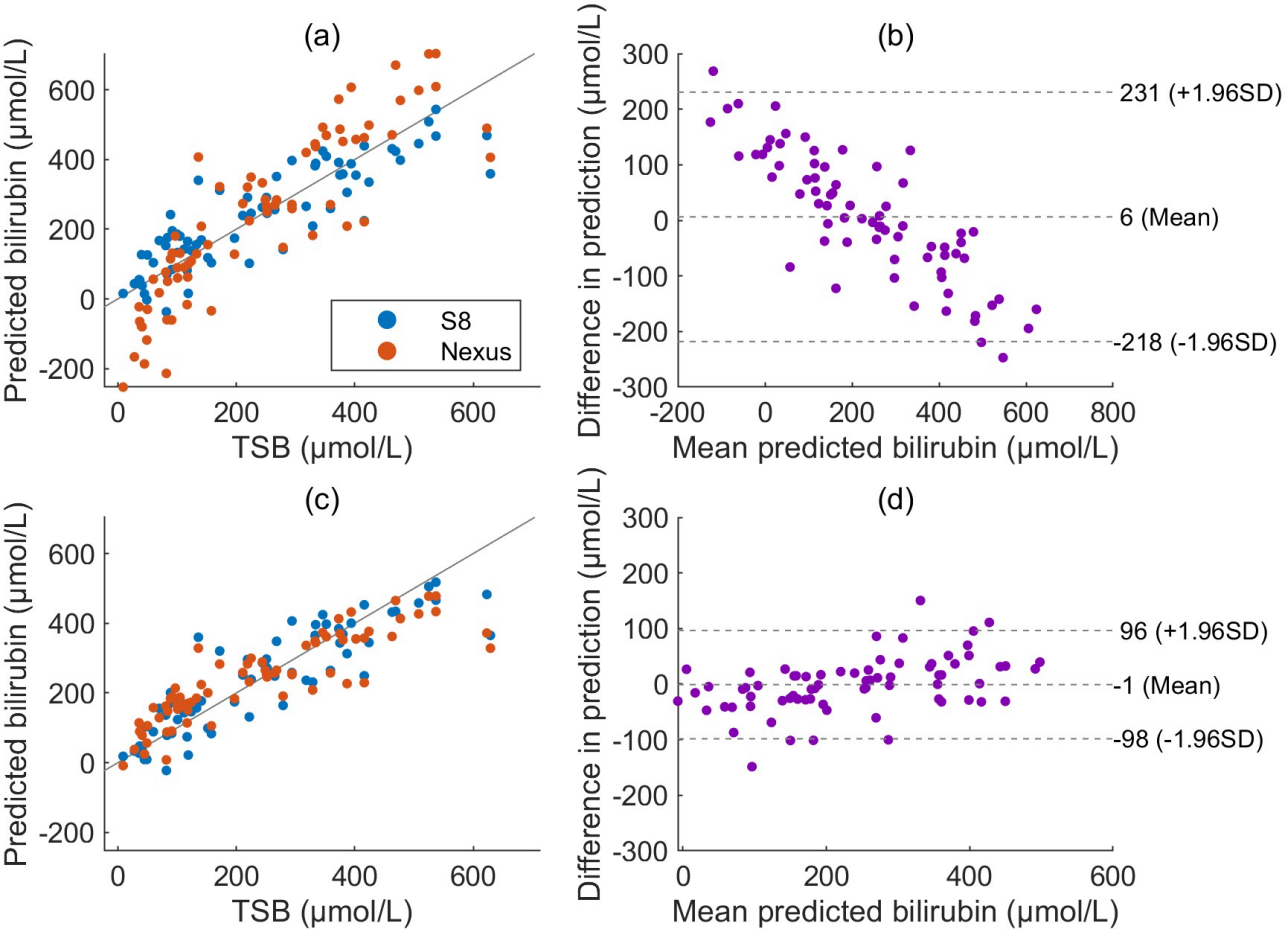
Importance of accounting for device-dependence. The predicted bilirubin values are plotted as a function of the known bilirubin levels (TSB) for the sclera site using a regression based on the S8 phone images before (a) and after (c) applying the device-specific color mappings (n = 72). Corresponding Bland-Altman plots for the agreement of the two phones to one another are shown in (b) and (d).

Fig 4 demonstrates the importance of accounting for device dependence when using the two-step method outlined in the second image capture methods section. Two models converting sclera color to predicted TSB were developed. The first was developed after applying ambient subtraction, thus standardising the lighting, but without applying the device-specific color mapping. This situation often occurs in pilot studies, where care is not taken to remove device dependence. The second model was developed after applying the color mapping, whereupon data should be independent of the lighting and device used for image capture. The results from the S8 are shown in blue in Fig 4. The difference in accuracy with and without the color mapping is negligible for this phone.

We then consider the situation where further data is collected using a different phone—the Nexus. If the first model is used, predicted TSB levels for the Nexus are extremely different from both the S8 and ground truth TSB. This is highlighted by the large number of negative predicted TSB values shown in red in Fig 4(a), and the strong trend observed in the Bland Altman plot in (b). Without the one-time calibration step, an entirely new set of patient data would need to be collected using the Nexus phone (or any other new device) in order to obtain reliable predictions. However, when device dependence is accounted for both in model development and Nexus processing, the mean squared error of the predicted bilirubin compared to TSB is no longer significantly different between the two phones (t-test, p = 0.34, df = 71). This is demonstrated in Fig 4(c) and 4(d), noting the 50% reduction in the 95% confidence intervals in (d) of -98 to 96*μ*Mol/L compared to (b) with -218 to 231*μ*Mol/L. When the method of [6] is followed, it is, therefore, possible to collect just one set of paired phone images and TSB values to develop a model, and then use this model to obtain reasonable predicted values using a different phone, and, in the case of the Nexus phone, a different camera (front) and illumination (white screen).

### Comparison of different imaging sites

**Fig 5:**
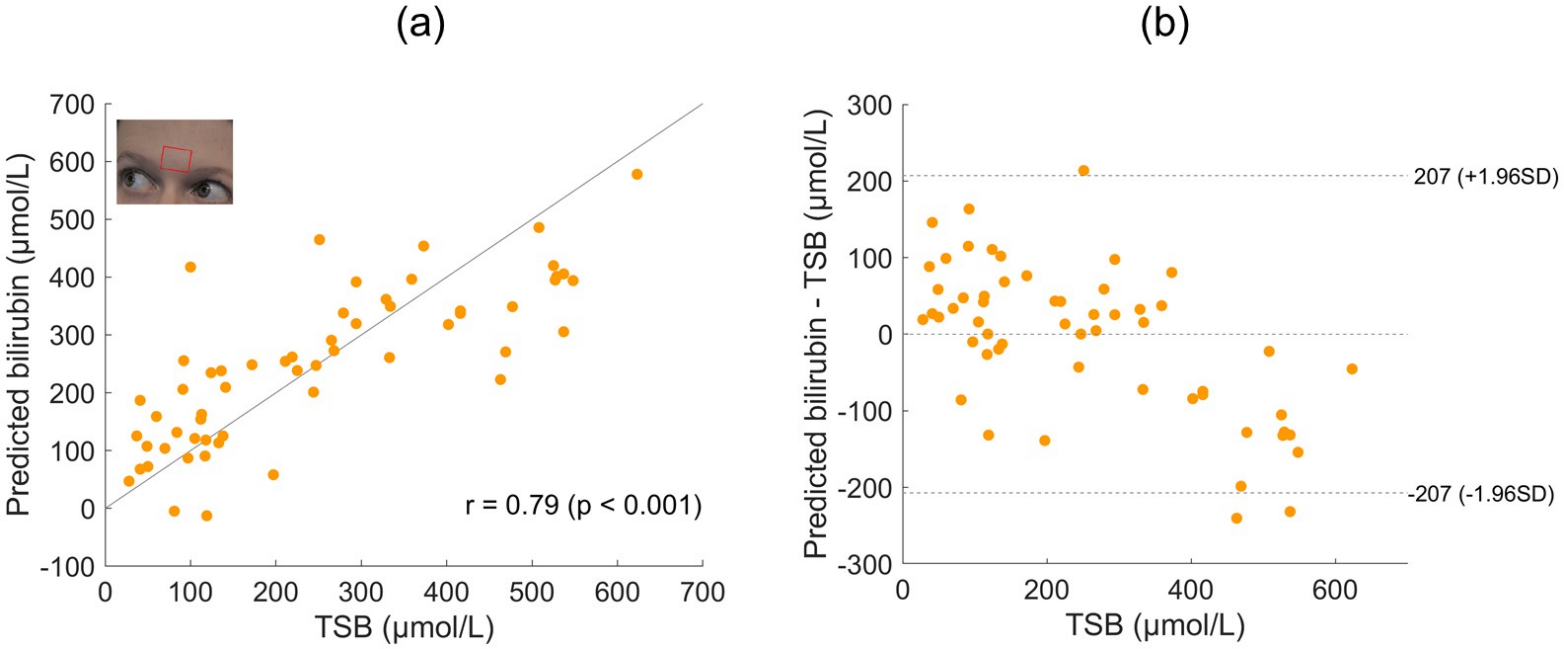
Forehead imaging site. (a) Predicted bilirubin levels are shown against the measured total serum bilirubin level (TSB) for the S8 phone using the forehead imaging site. The Pearson linear correlation coefficient is displayed (n = 57). (b) Bland Altman plot for the data presented in (a).

**Fig 6:**
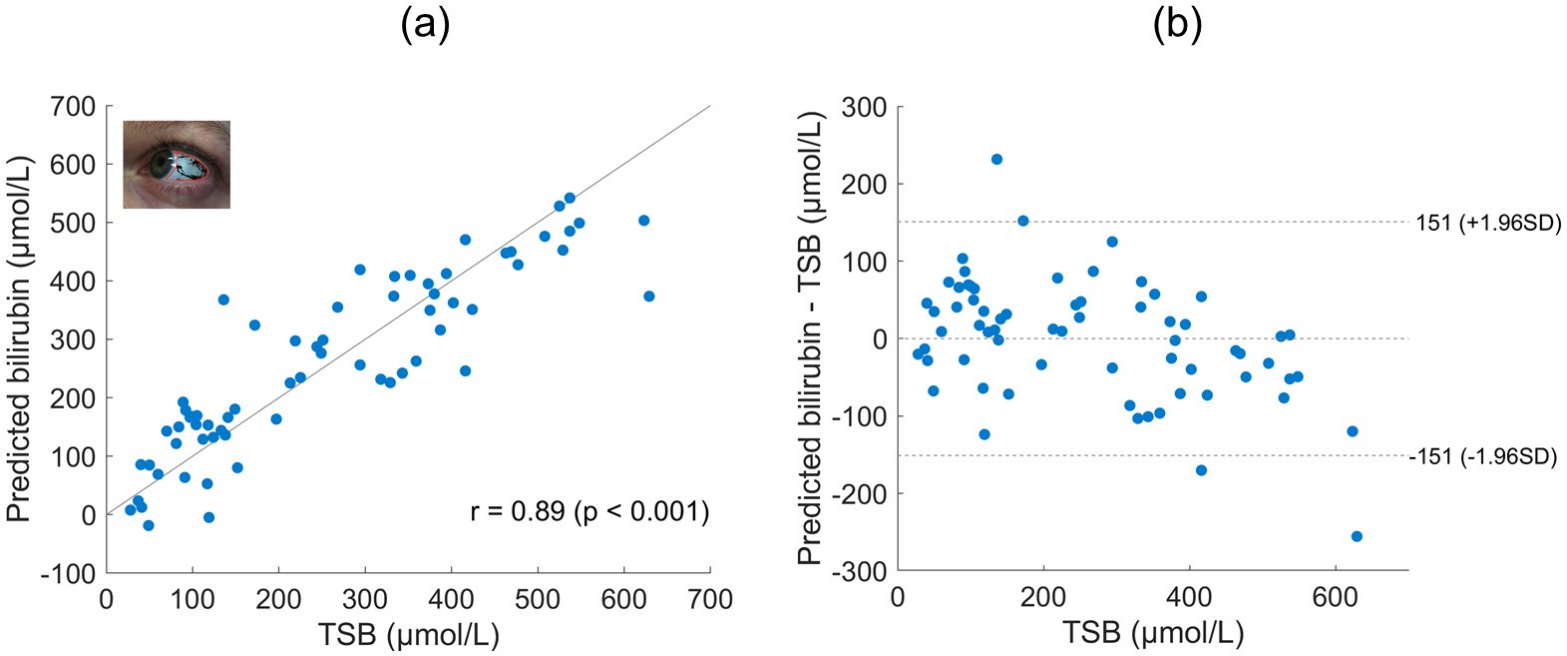
Sclera imaging site. (a) Predicted bilirubin levels are shown against the measured total serum bilirubin level (TSB) for the S8 phone using the sclera imaging site. The Pearson linear correlation coefficient is displayed (n = 66). (b) Bland Altman plot for the data presented in (a).

**Fig 7:**
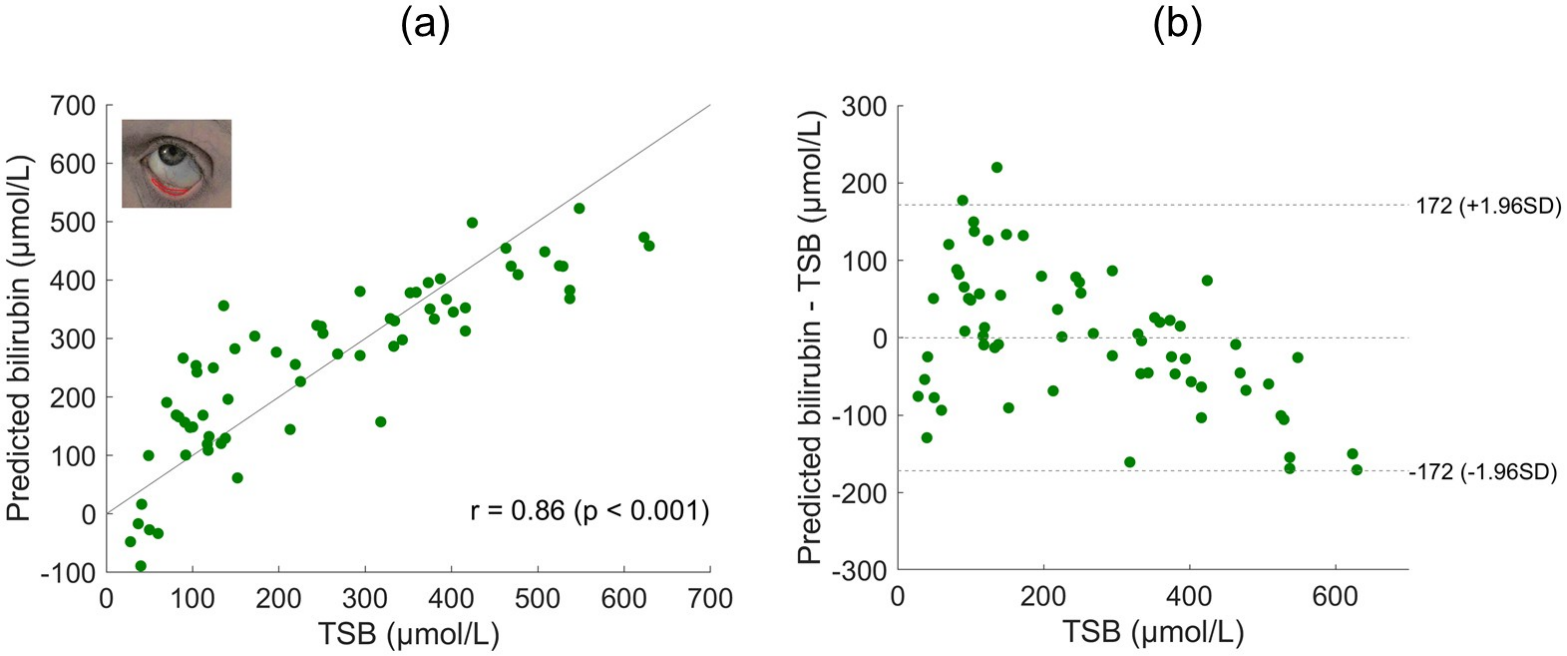
Lower eyelid imaging site. (a) Predicted bilirubin levels are shown against the measured total serum bilirubin level (TSB) for the S8 phone using the lower eyelid imaging site. The Pearson linear correlation coefficient is displayed (n = 66). (b) Bland Altman plot for the data presented in (a).

**Fig 8:**
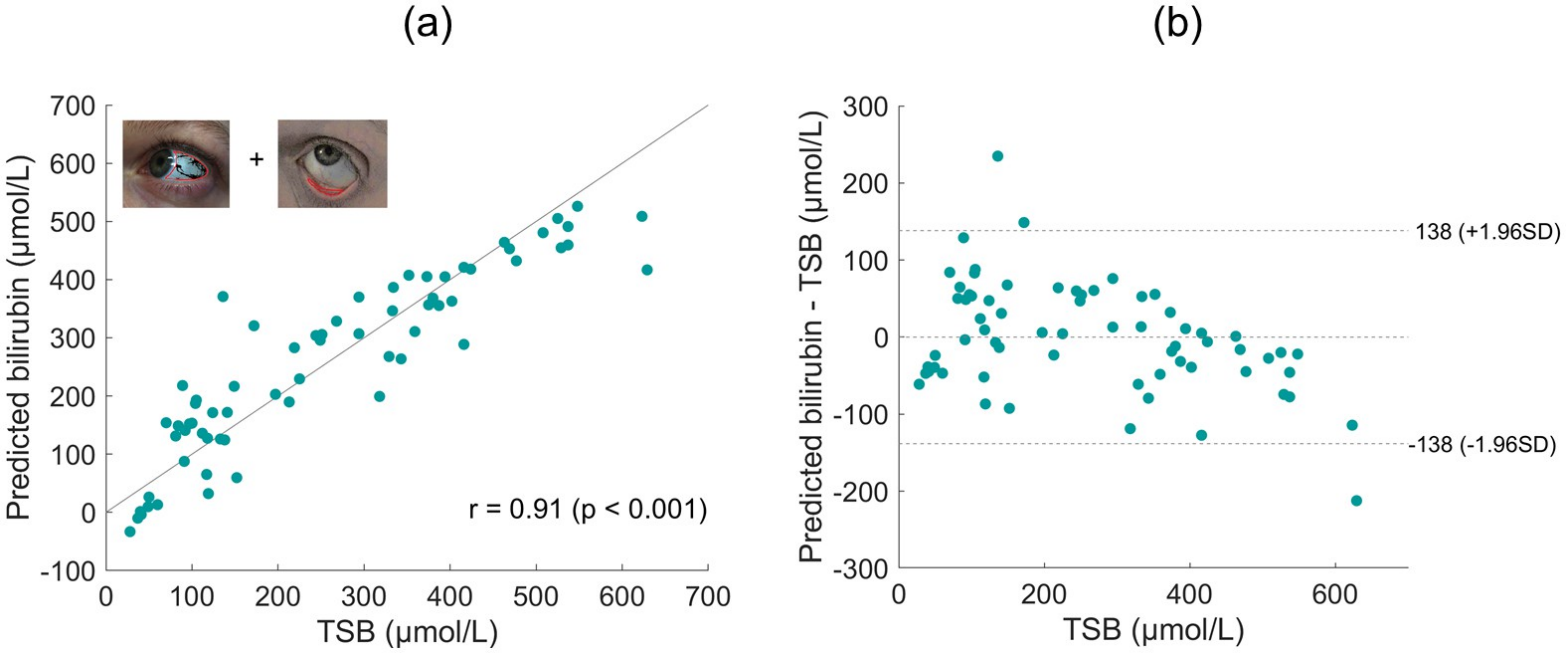
Combined sclera and lower eyelid imaging sites. a) Predicted bilirubin levels are shown against the measured total serum bilirubin level (TSB) for the S8 phone using data from both the sclera and lower eyelid imaging sites. The Pearson linear correlation coefficient is displayed (n = 66). (b) Bland Altman plot for the data presented in (a).

Having validated that the processing used accounts for variations introduced by ambient light and different devices, we focus on comparing results from different imaging sites. Data presented here is from a single phone S8 for clarity. Figs 5, 6, 7 and 8 present predicted bilirubin as a function of measured bilirubin and associated Bland Altman plots for models based on data from images of the forehead, sclera, lower eyelid and sclera and lower eyelid combined, respectively. The correlation of predicted and measured bilirubin, and the limits of agreement of the Bland Altman plots are used to assess the quality of the results.

The results from Fig 5 using the forehead are better than anticipated based on previous work [3, 14]—since no correction for melanin was performed it was expected that the performance would be affected. However, the input images were highly biased, with 80% of Caucasian patients, 15% of Asian patients and just 5% of Black patients. This bias likely explains the good performance observed with this dataset, and these results alone should not be taken as an endorsement of extracting color from the skin without attempting to correct for melanin.

The best results using a single imaging site are yielded from the sclera, as shown in Fig 6. The lack of melanin in the sclera will have helped, however the reduced size and reflective nature were challenges. Patients were largely able to comply with instructions to look in a particular direction to expose more of the sclera. Additionally, applying the filtering approach detailed in the Method section removed the majority of pixels affected by reflection and containing blood vessels.

Results based on the lower eyelid, shown in Fig 7, are similar to those from the sclera. In fact, the mean squared error of the predicted bilirubin compared to TSB is not significantly different when using the sclera or lower eyelid as the ROI (t-test, p = 0.18, df = 65). This is a surprising result as the change in color to the naked eye is far less pronounced in the lower eyelid than the sclera. Image capture for the lower eyelid dataset was more challenging than for the sclera, since the process of sufficiently pulling down the eyelid was less comfortable for patients and more prone to error than simply looking to one side. Given their similar results and the easier image capture, the sclera is clearly the better imaging site for this application.

Rather than generating a model for bilirubin based on just one imaging site, a multivariate approach is possible [15]. Fig 8 presents results generated by combining data from the sclera and lower eyelid. Whilst currently images are captured separately for the sclera and lower eyelid to maximise the quality of both, it would be possible to extract the scleral color from one single lower eyelid image. However, as discussed above, for clinical translation and independent image capture, use of the lower eyelid would have low patient acceptability.

## Discussion and conclusions

The main work focussing on bilirubin in an adult population prior to this was published by Mariakakis et al [8], which aimed to screen for very early-stage jaundice as a biomarker for pancreatic cancer. Scleral images of 70 patients were collected using a single phone, and a headset-style box was used to block all ambient illumination and allow the phone to provide the lighting. A machine learning approach was used to obtain bilirubin estimates, yielding a 0.89 correlation.

In this paper, we have demonstrated the use of two processing methods to enable assessment of bilirubin levels in patients with advanced cirrhosis and a wide range of TSB, including bilirubin values 20-30 times the upper limit of normal range, accounting for both lighting and device dependence. Image data was corrected either using a per-image calibration based on inclusion of a color chart in each image, or using the two-step process from [6] based on ambient subtraction of flash/ no-flash image pairs and a one-time calibration thus avoiding the need to include a color chart in each image.

We have investigated which imaging site is best for patients with cirrhosis between the forehead, sclera and lower eyelid. The forehead yielded the poorest results, owing to the confounding factor of melanin. We found that using the sclera or lower eyelid yielded similar strong results, with linear correlation coefficients of 0.89 and 0.86, respectively (p<0.001). For alternative applications where lower eyelid images are already being captured, this finding is highly relevant as the lower eyelid has not been previously considered as an imaging site for monitoring jaundice. For assessing bilirubin levels in patients with cirrhosis, owing to the simpler image capture for the sclera, we recommend the use of the sclera as the imaging site. See our clinical paper presenting results based on the scleral imaging site and how they correlate with clinical scores such as Clinical Liver Failure Consortium Acute-Decompensation score and grade of Acute-on-Chronic Liver Failure [16]. The very strong correlation of predicted bilirubin via the sclera with TSB even at values greater than 20x the upper limit of normal, indicates promising clinical utility for real-world application. To further improve the usability for both patients and clinicians, this approach for non-invasive assessment of bilirubin could be incorporated into an app enabling monitoring alongside other key biomarkers.

Cirrhosis patients need on-going monitoring for signs of decompensation. In this paper, we have not evaluated the diagnostic accuracy of the technique on the same patients longitudinally. Further investigation in this area will be carried out to establish the full potential of this imaging technique in home monitoring.
